# NGS-PrimerPlex: High-throughput primer design for multiplex polymerase chain reactions

**DOI:** 10.1371/journal.pcbi.1008468

**Published:** 2020-12-30

**Authors:** Andrey Kechin, Viktoria Borobova, Ulyana Boyarskikh, Evgeniy Khrapov, Sergey Subbotin, Maxim Filipenko

**Affiliations:** 1 Institute of Chemical Biology and Fundamental Medicine, Siberian Branch of the Russian Academy of Sciences, Novosibirsk, Russia; 2 Novosibirsk State University, Novosibirsk, Russia; Johns Hopkins University, UNITED STATES

## Abstract

Multiplex polymerase chain reaction (PCR) has multiple applications in molecular biology, including developing new targeted next-generation sequencing (NGS) panels. We present NGS-PrimerPlex, an efficient and versatile command-line application that designs primers for different refined types of amplicon-based genome target enrichment. It supports nested and anchored multiplex PCR, redistribution among multiplex reactions of primers constructed earlier, and extension of existing NGS-panels. The primer design process takes into consideration the formation of secondary structures, non-target amplicons between all primers of a pool, primers and high-frequent genome single-nucleotide polymorphisms (SNPs) overlapping. Moreover, users of NGS-PrimerPlex are free from manually defining input genome regions, because it can be done automatically from a list of genes or their parts like exon or codon numbers. Using the program, the NGS-panel for sequencing the *LRRK2* gene coding regions was created, and 354 DNA samples were studied successfully with a median coverage of 97.4% of target regions by at least 30 reads. To show that NGS-PrimerPlex can also be applied for bacterial genomes, we designed primers to detect foodborne pathogens *Salmonella enterica*, *Escherichia coli* O157:H7, *Listeria monocytogenes*, and *Staphylococcus aureus* considering variable positions of the genomes.

## Author summary

The polymerase chain reaction is an extensively applied technique that helps to identify bacterial and viral pathogens, germline and somatic mutations. And primers are one of the most important parts of each PCR-assay, defining its sensitivity and specificity. Many tools have been developed to design primers automatically, however, none of them let someone develop multiplex PCR assays requiring to design from two to thousands of primers with one-two commands. Here we present such a tool called NGS-PrimerPlex that was initially developed to create new targeted NGS-panels. This approach has become widespread since it allows targeting only the regions of interest of a genome reducing the analysis cost. We showed that NGS-PrimerPlex can be applied for the multiplex pathogen detection and sequencing exons of the *LRRK2* gene in Parkinson’s disease patients. The primers designed were validated on 354 DNA samples for which the *LRRK2* coding sequences were successfully studied on the MiniSeq Illumina platform.

This is a *PLOS Computational Biology* Software paper.

## Introduction

Multiplex polymerase chain reaction (PCR) is a fundamental approach to get many several fragment copies of a single DNA molecule simultaneously. It has a lot of applications in different science areas, from kinship determination [1] and pathogen detection [2] to next-generation sequencing (NGS) library preparation [3,4]. The primer design process involves two main steps. 1) Choosing target regions, while a researcher converts gene names into sequences. 2) For each target region, primers should be constructed taking into consideration a lot of parameters (amplicon and primer lengths, melting temperature, whole primer and only 3’-end GC-content, poly-N tract presence, secondary structures: homo- and heterodimers, hairpins, hybridization to non-target regions, overlapping variable positions). However, a transition from monoplex to multiplex reactions requires consideration of more factors that may influence PCR efficiency (secondary structure formation by oligonucleotides and non-target hybridization, elongation, and amplification with primers from different pairs). The number of factors to consider grows exponentially with the number of target sequences that make the manual primer design labor-intensive.

To facilitate the primer design process, many tools have been developed (**S1 Table**). However, none of them can completely simplify the process of primer design considering all factors listed above, particularly for developing new amplicon-based NGS panels, that require additional checking for secondary structures due to the adapter sequences at the 5’-ends of each primer. At the same time, the amplicon-based NGS panels have become widespread among both researchers and commercial companies due to its simplicity of utilization by end-user and highly efficient target enrichment for DNA samples of different quality, including to detect somatic mutations with a low tumor frequency. And two specific approaches are used to increase the yield of a genome region studied (nested PCR) or to get all possible sequences of a variable region (anchored PCR). Nested multiplex PCR is applied in molecular biology where higher specificity is necessary [5], e.g. during the detection of low-frequent somatic mutations in tumors [6] or pathogen identification analyzing conservative sequences with enough variability [7]. The use of the nested PCR increases the assay specificity by amplifying only regions that contain both internal and external primers (**S1 Fig**) that also increases the number of parameters to analyze. The anchored PCR was developed to study rare transcripts with sequence unknown partially in 1988 [8], however, recently, it has gained relevance again due to the need to detect particular gene fusions with an unknown partner [9]. And this type of PCR requires the program to be able to design primers on one side only that is commonly not supported by other primer design tools.

Here we describe a new tool for automatic primer design for multiplex PCR, including for creating new amplicon-based targeted NGS-panels, NGS-PrimerPlex. The program is implemented in Python, takes into consideration all parameters listed above, and was tested on different sets of target sequences, including bacterial and human genomes. Primers designed with NGS-PrimerPlex were validated experimentally for studying the *LRRK2* gene coding sequences.

### Design and implementation

NGS-PrimerPlex is implemented in Python using free-available Python-modules (**S2 Table**) and the BWA program [10]. It can be run as a standalone program (only for macOS and Linux users) or inside a docker image with or without a graphical user interface (GUI) (for users of any OS, **S2 Fig**). Docker image allows someone not to install and not to download additional files but use it immediately after downloading the main package. NGS-PrimerPlex and its manual with screenshots are available at the GitHub server (https://github.com/aakechin/NGS-PrimerPlex, https://github.com/aakechin/NGS-PrimerPlex/wiki) and in the S1 Text.

### NGS-PrimerPlex implementation

#### Getting genome coordinates based on a list of genes and their parts

**Fig 1:**
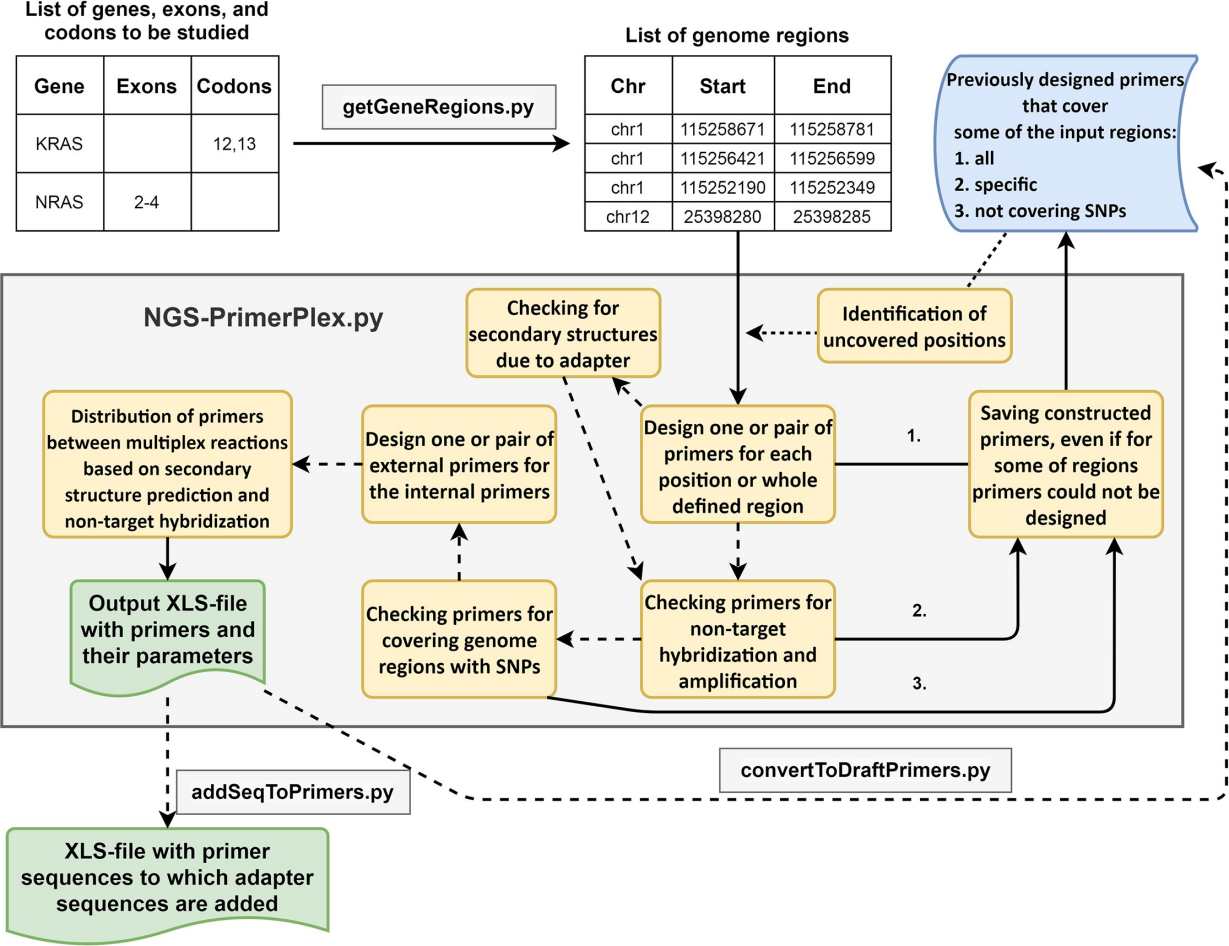
The whole workflow of the NGS-PrimerPlex. Solid arrows denote obligatory steps; dotted arrows denote optional steps that can be applied by the user. The primer design process starts from the list of sequence regions (e.g. regions of a reference genome), however, such region coordinates can be automatically obtained with getGeneRegions.py script. NGS-PrimerPlex tries to design primers for all input regions saving primers constructed to the XLS-file (“draft” file 1). If it is necessary, all primers are verified for the forming secondary structures due to the additional adapter sequences at the 5'-ends of primers. If all target regions are covered, the primers are verified for non-target hybridization and amplification. Primers that do not hybridize to many targets and do not form non-target amplicons are saved to distinct XLS-file (“draft” file 2), others are removed. If all target regions are still covered, the primers are verified for overlapping genome regions with variable positions. Primers that do not overlap variable positions with defined parameters (the frequency of the alternative allele and the distance to the 3'-end) are saved to the new XLS-file (“draft” file 3), others are removed. If all targets are still covered, primers are distributed to the defined number of pools. The diagram was drawn in https://app.diagrams.net.

NGS-PrimerPlex contains script getGeneRegions.py that reads the table of genes from the input file. For each input gene, the chromosome is determined from the comma-separated values (CSV) file that is created automatically from the reference genome GenBank- and FASTA-files. Then exon/codon coordinates are extracted from the corresponding GenBank-file if it is required. User can specify numbers of exons or codons which are necessary to be included. Human reference genome versions hg19 or hg38 or reference genome of other organisms can be used. The output file can be used in the next script for the primer design NGS-primerplex.py. Whole NGS-PrimerPlex package functionalities are reflected in **Fig 1**.

#### Multi-step primer design

By default, NGS-PrimerPlex splits input genome regions onto distinct positions and tries to construct primers for each of them using primer3-py that includes the primer3 library [11]. For each position, the program tries to design three types of primers: so that the right primer was close to the studied position, the left primer was close to the studied position, and without any of these restrictions (**S3 Fig**). This is necessary for subsequent successful joining of primers into a set of primer pairs that amplify a single extended genome region (e.g. whole exon). Unlike other programs (e.g. hi-plex) that design primer pairs not overlapping at all, NGS-PrimerPlex allows overlapping but considers it during distribution into multiplex reactions. This increases the chances of successful primer design, particularly for genome regions with complex structures. At the same time, NGS-PrimerPlex allows users to forbid splitting of some regions, e.g. during primer design to detect *EGFR* exon 19 deletion [12].

Another feature of the NGS-PrimerPlex that may be useful for complex genome regions is a multi-step primer design. Frequently, existing tools suggest users choose parameters of primer design and at the end of the design process program can give an error that primers could not be constructed with the defined parameters. After choosing less strict parameters, the user has to start the process from scratch that significantly slows down parameter optimization. NGS-PrimerPlex saves primers that could be designed with more strict parameters and constructs primers only for other regions. On the other hand, this feature can be used to create personalized NGS-panels and expanding existing ones that are targeted for patient-specific genome regions, when primers for some of these regions have been obtained earlier that can not be done with other programs.

#### Non-target hybridization in the genome and overlapping with SNPs

On-target is one of the main characteristics of targeted NGS-panels [13] and it depends on whether the primers hybridize to non-target genome regions, including substitutions/insertions/deletions as well as with other primers from the same multiplex reaction. For doing it, NGS-PrimerPlex uses the BWA program that can readily find all regions to which a nucleotide sequence can be mapped. For the similar regions (but not necessarily identical ones, because the user can search for primer targets with mismatches), the program checks if the primer has the same last two nucleotides from 3’-end as the non-target region, that is not performed by existing tools (e.g. MPD and hi-plex). This allows identifying regions that will give non-target amplicons but not only have homology with primers. Comparing genome coordinates for different primers, NGS-PrimerPlex filters out primer pairs that can lead to non-target amplification in a multiplex reaction.

Another significant characteristic of NGS-panels is uniformity of coverage [13] which depends, among other things, on the sequence homogeneity of the sequence complementary to primer among different samples. Therefore, NGS-PrimerPlex checks if any of the primers designed overlaps with SNPs from the dbSNP database [14] using the pysam Python-module and dbSNP variant call format (VCF) file. The user has an opportunity to check only part of the primer for overlapping with SNP (e.g. only the last 10 nucleotides) and to define the minimal frequency of SNP in the population for which primers will be checked. Both features are unique for NGS-PrimerPlex.

#### Nested and anchored multiplex PCR

NGS-PrimerPlex allows users to design primers for nested PCR with subsequent distribution of four primers among multiplex reactions considering both secondary structure and non-target product formation for internal and external primers.

Anchored multiplex PCR that applies one primer hybridizing to the gene-specific region and one primer been complement to the adapter sequence allows amplifying regions with unknown or partially highly variable sequences (**S1 Fig**), e.g. to detect gene fusion mutations without prior knowledge of the fusion partners [6,15]. And NGS-PrimerPlex can also design primers for this type of multiplex PCR.

#### Distribution of primers among multiplex reactions

The final step of the primer design is the distribution of the constructed primers among the user-defined number of multiplex reactions. NGS-PrimerPlex supports distribution of all primers into any number of multiplexes (unlike e.g. MPD that is capable of distributing into small multiplexes of about 2–15 primer pairs) as well as the distribution of some regions into specific groups of multiplexes (e.g. when it is necessary to separate some genes into different multiplexes). The distribution is performed using the networkx Python module which creates a graph, edges of which mean two primer pairs not producing any secondary structures, not overlapping by the product, and not producing non-target amplicons. For joining primer pairs into a set of primer pairs that amplify a single extended genome region (e.g. whole exon), it searches for the shortest path from the start of such region to the end, both of which are also included into the graph. For the subsequent distribution, NGS-PrimerPlex tries to find a clique in the constructed graph, i.e. such a subset of vertices that every two distinct vertices in the clique are adjacent. Searching for clique is performed until all primer pairs are distributed.

### NGS-PrimerPlex throughput

To evaluate NGS-PrimerPlex throughput, we designed primers for studying *BRCA1* and *BRCA2* coding sequences. Coding exon regions sorted by the length were added one by one to the NGS-panel and the computer time was registered.

### Primer design for the bacterial pathogen detection

**Fig 2:**
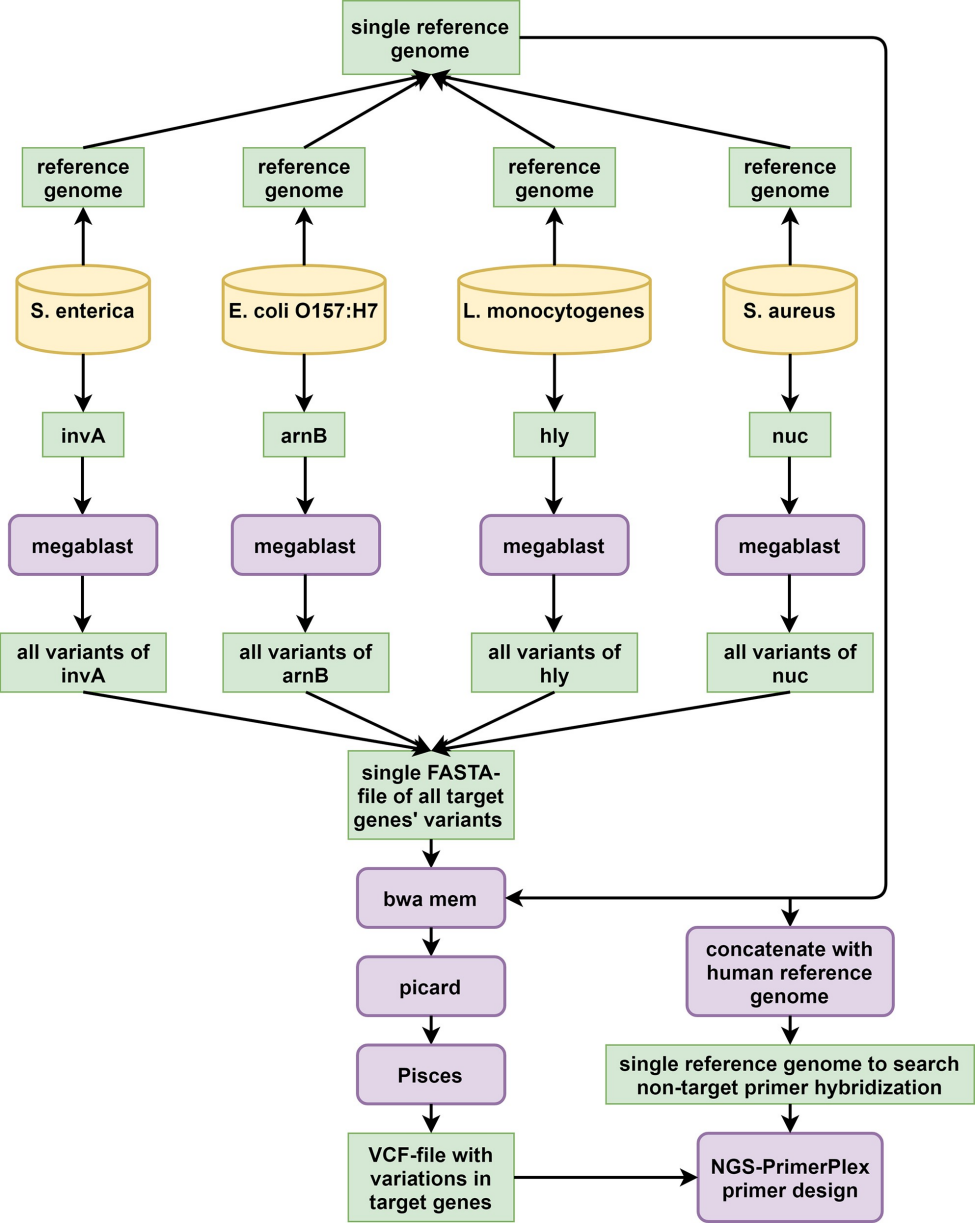
The primer design workflow for the foodborne bacterial pathogen detection.

To demonstrate NGS-PrimerPlex application for bacterial and viral species identification and genome analysis, we designed multiplex PCR primers that targeted *invA*, *arnB* (not *rbfE* as it is called incorrectly in the cited article), *hly*, and *nuc* genes of *Salmonella enterica*, *Escherichia coli* O157:H7, *Listeria monocytogenes*, and *Staphylococcus aureus*, respectively (**Fig 2**) [2]. We downloaded four reference genomes for these species in FASTA and GenBank formats and combined FASTA-sequences into one artificial reference-file. The file with target regions was based on the gene coordinates. We tried different single positions of these genes as targets, while the primers designed did not meet all requirements. To identify variable positions in the target genes, sequences of the target genes were extracted and using the megablast algorithm of NCBI (https://blast.ncbi.nlm.nih.gov/), we found all similar sequences for them with filter by the query coverage 90%. Found sequences were also joined into one FASTA-file and were mapped onto the artificial reference file with BWA. SAM-file was converted into BAM-file with Picard (http://broadinstitute.github.io/picard), and variations were called with Pisces (https://github.com/Illumina/Pisces) (—minbq 1,—minmq 10,—sbfilter 10,—minvq 0,—minvf 0.0005, -c 1). The VCF-file obtained was archived with the bgzip and indexed with the tabix commands. To exclude primers that target any human genome regions, human chromosome sequences were also added to the artificial reference genome file, and primer design with NGS-PrimerPlex (-maxampllen 300, -primernum1 3, other parameters were default) was carried out.

### NGS library preparation and sequencing the *LRRK2* gene

To confirm the efficiency of NGS-panels designed with the NGS-PrimerPlex tool, we applied it to sequence coding exons of the *LRRK2* gene, associated with Parkinson’s disease [16]. The *LRRK2* gene includes 51 exons, all of which are protein-coding (7784 bp including two intron nucleotides near exon-intron junctions). DNA was extracted from the peripheral blood leukocytes as was described earlier [17]. Patient consents have not been obtained, because data were analyzed anonymously. NGS libraries were prepared with an in-house amplicon-based approach using two-step amplification: (1) enrichment of target regions; (2) inclusion of adaptors. The libraries were sequenced with the MiniSeq High Output kit (300 cycles). NGS-reads were analyzed with a workflow that is similar to BRCA-analyzer’s one [17]. NGS-reads were trimmed with Trimmomatic [18] and mapped to the human reference genome (hg19). Coverage was evaluated with samtools [19] and Python-scripts. Variations were called with Pisces (https://github.com/Illumina/Pisces).

## Results

### Developing the program

NGS-PrimerPlex has many features that are useful during developing new NGS-panels (**S1 Table**). Among them, we note the following features: (1) choosing genome regions based on the list of genes and their parts (list or range of exon and/or codon numbers); (2) multi-step primer design that allows efficient optimizing of primer design parameters (GC-content, Tm, amplicon length, etc.); (3) additional checking primers for forming secondary structures due to adapter sequences at the 5'-end of primers; (4) checking primers for non-target hybridization in the genome; (5) checking primers for overlapping with variable genome sites, e.g. single-nucleotide polymorphisms (SNPs); (6) designing primers for nested PCR; (7) designing primers for anchored PCR when target region is amplified from one sequence-specific primer and one primer that is complementary to an adapter sequence; (8) designing primers for whole defined genome region; (9) automatic distribution of primer pairs between multiplex reactions based on secondary structures and non-target amplicons that can be formed by primers from different pairs.

### NGS-PrimerPlex throughput

**Fig 3:**
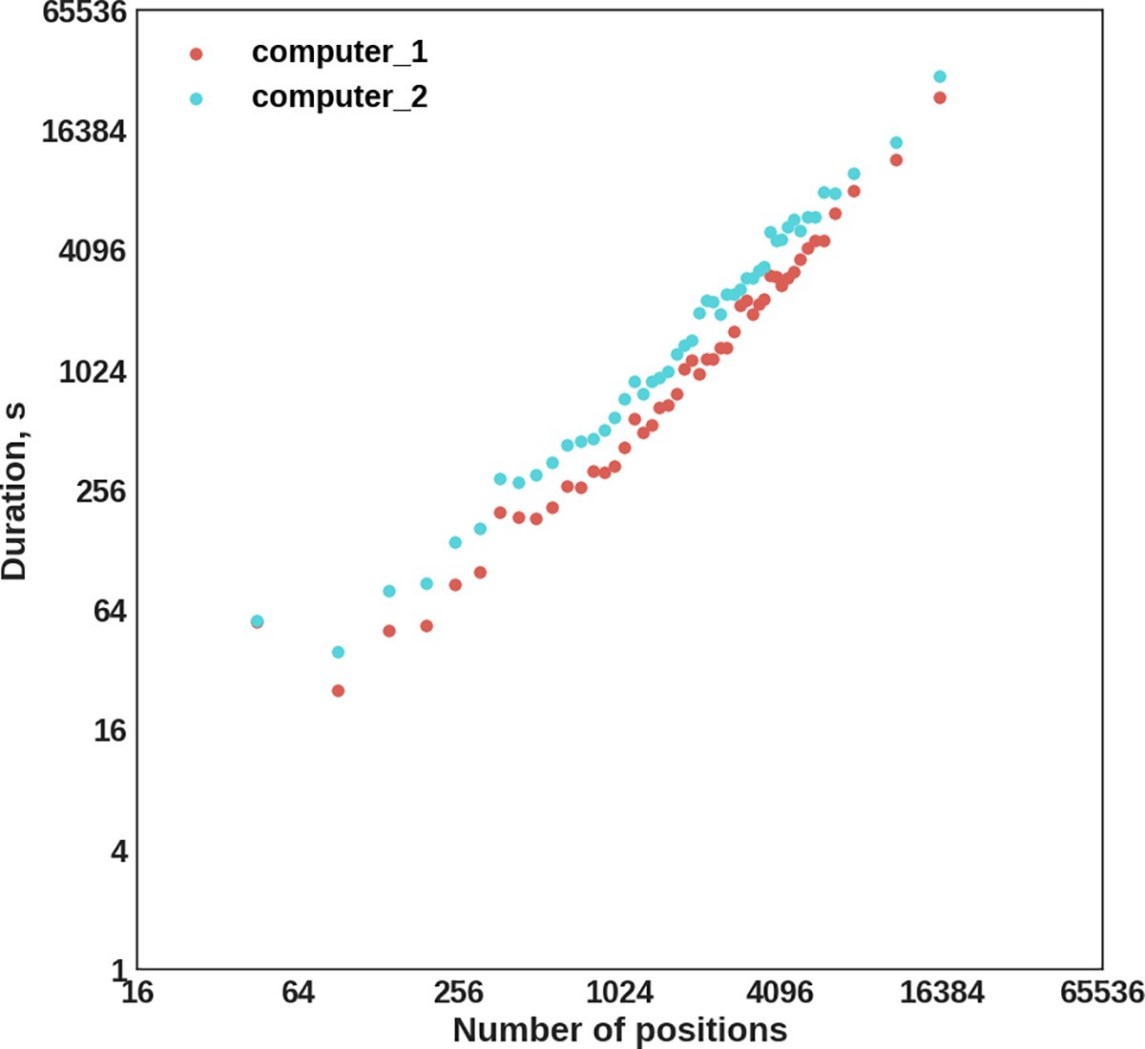
The NGS-PrimerPlex performance evaluated for the different numbers of *BRCA1* and *BRCA2* exons on two computers (computer 1: Intel Core i7-8700 3.2GHz, 64 GB RAM, Ubuntu 16.04; computer 2: Intel Core i7-2700K 3.5GHz, 32 GB RAM Ubuntu 18.04). Horizontal and vertical axes have log_2_-scale.

To evaluate the throughput of the NGS-PrimerPlex, we applied it to design primers for the different number of *BRCA1* and *BRCA2* coding exons sorted by the length (**Fig 3**). The computer time of the primer design was almost equal to the number of positions covered.

### NGS-PrimerPlex for detecting bacteria and viruses

NGS-PrimerPlex can be used to design primers to detect bacteria and viruses or to study their genomes (e.g. to sequence coronavirus genome with an amplicon-based NGS-panel). To demonstrate it, we designed primers to detect four foodborne bacterial pathogens (**Table 1**). While constructing, we took into consideration two important potential obstacles: 1) primers can give amplicons not only from the target genome but also from the human genome, leading to false-positive results; 2) primers can overlap with variable positions of the target gene, that may lead to false-negative results. For the primers described in the literature [2], we found 12 variable positions overlapped by primers with the population frequency 2–64% (**Table 1**). Primers designed with the NGS-PrimerPlex did not overlap variable positions with the alternative allele frequency of more than 1%. Another way to avoid variable positions under primers is the use of the target region as a whole if the alignment has been carried out earlier. In the NGS-PrimerPlex this can be turned on by writing ‘W’ in the 7^th^ column of the regions file. This can be useful for sequencing the most variable parts of genes among different species of one genus or different isolates of one species.

**Table 1:** Primers for the detection of foodborne bacterial pathogens. Variable positions in primers from the literature are in bold and underlined. The second variable position for the first *S*. *enterica* primer has two alternative alleles (T and A).

Organism	Primers from the literature and the alternative allele frequency	NGS-PrimerPlex primer
*S*. *enterica* (*invA*)	CCAG**C**CGTCTTAT**C**TTGA 23%–T; 2%–T, 2%–A	CGAGATCGCCAATCAGTCC
	CATCGCACCGTCAAA**G**, 2%–A	TTAAATTCCGTGAAGCAAAACGT
*E*. *coli O157*:*H7 (arnB)*	CAGTTTACCAACCGTCATT, No	GGGTGCTTTTGATATTTTTCCGA
	GG**T**GGCTCCTGTGTATTTTA, 1%–C	GGAAAGAGAGGAATTAAGGAATCAC
*L*. *monocytogenes (hlyA)*	GCCGTAAG**T**GGG**A**AATC, 50%–C, 50%–G	CGAAAAGAAACACGCGGATGAAA
	ATA**G**GCAATGGGAACTCC, 64%–A	CGAAAAGAAACACGCGGATGAAA
*S*. *aureus (nuc)*	**C**GGCGTAAATAGAA**G**TG**G**, 47%–T; 2%–T; 3%–A	TTCAAGTCTAAGTAGCTCAGCA
	C**C**GTATCACCATCAATCG, 12%–A	ATGTAATTTTTTAGTTGAAGTTGCACT

### NGS library preparation and sequencing the *LRRK2* gene

**Fig 4:**
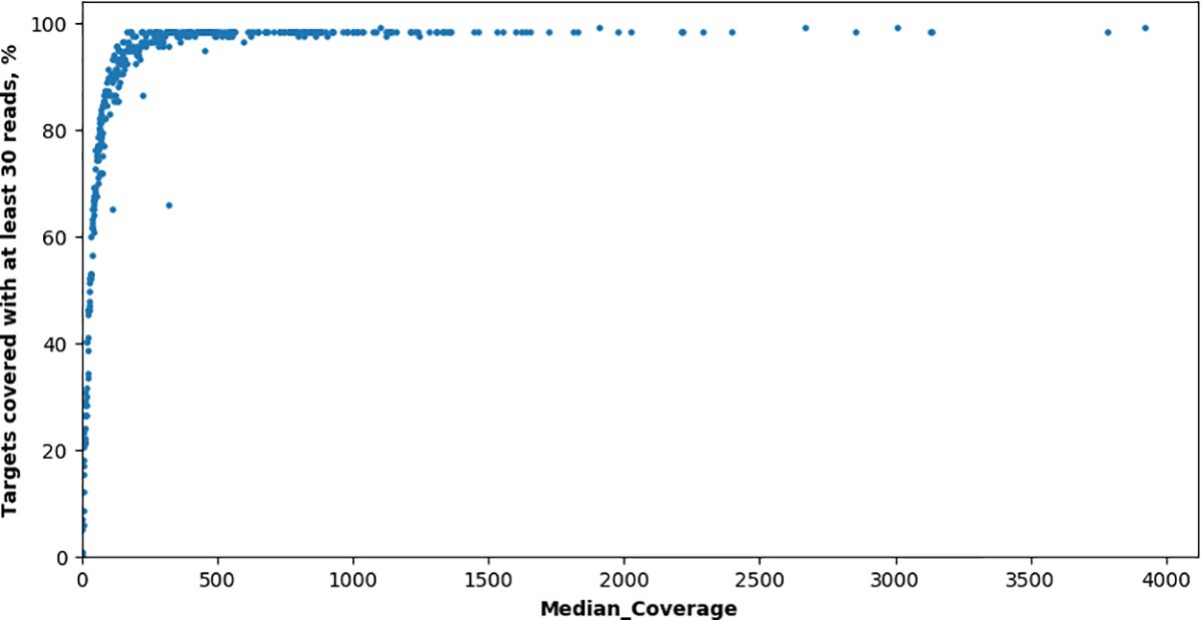
Percent of targets covered by at least 30 reads vs median coverage for samples sequenced for the *LRRK2* gene. Each dot is a distinct DNA sample.

117 primer pairs were automatically designed without checking primers for covering SNPs (to evaluate their effect depending on remoteness from the 3’-end of primer) and sorted to three multiplex reactions (each of 39 primer pairs) with NGS-PrimerPlex to sequence coding regions of the *LRRK2* gene (**S3 Table**). 403 DNA samples from 403 patients were analyzed with the created NGS-panel (the NGS reads are available at the NCBI Sequence Read Archive with the BioProject accession number PRJNA664536). For samples with a median coverage of target regions by at least 30 reads (354 samples), the median percent of targets covered by at least 30 reads was 97.4% (**Fig 4**). For other samples, the median coverage of target regions was less than 30, likely due to low amounts or low quality of DNA used. One amplicon was uncovered, one amplicon was covered by at least 30 reads only for five samples, three amplicons had a high variation in the coverage between samples due to overlapping with SNPs (this NGS-panel was developed without checking for covering SNPs).

## Availability and future directions

NGS-PrimerPlex is a high-throughput cross-platform tool for automatic primer design for different multiplex PCR applications, including the creation of new targeted NGS-panels, and freely available at https://github.com/aakechin/NGS-PrimerPlex/. It involves many useful features that significantly facilitate the primer design process and are not supported or have limited use by existing tools for the multiplex PCR primer design (automatic target region extraction; multi-step primer design; secondary structures due to adapter sequences; non-target hybridization and amplicons for one-pair primers and primers from the same pool; overlapping with variable positions; nested and anchored PCR; primers for the whole region; automatic sorting to primer pools). The NGS-PrimerPlex throughput is enough to create targeted NGS-panels of different sizes, however, for some regions, the primer design process still requires high operator attention to select the best parameters or to choose a particular region to amplify targets with many pseudogenes in the genome (e.g. for *PMS2* and *CHEK2* genes) [20,21] or with high variability in species studied (e.g. for bacterial or viral genomes). And it can be a direction for future improvements.

## Supporting information

S1 Table Comparison of NGS-PrimerPlex and other tools functionality: **a**–choosing genome regions to amplify by gene names and their parts. **b**–primer design for all chosen genome regions. **c**–checking primers for non-target hybridization and non-target amplification. **d**–checking primers for covering variable genome sites that contain high-frequent SNPs. **e**–automatic primer distribution for several multiplex reactions, considering secondary structures and non-target amplicons that can be formed by primers from different pairs. **f**–primer design for nested PCR. **g**–primer design for anchored PCR when the target region is amplified from one sequence-specific primer and one primer complemented to the adapter sequence. It can be useful for the detection of gene fusions. **h**–has graphical or web-interface **yes/no**–means that this tool includes or does not include such functionality, respectively. ± –means that such functionality is partial (e.g. distribution among pools is restricted by a maximum of 15 primer pairs in one pool)**?** –means that such functionality was claimed but the tool wasn’t acceptable
(XLSX)

S2 Table List of Python-modules used in NGS-PrimerPlex. (XLSX)

S3 Table Primers designed with the NGS-PrimerPlex for the *LRRK2* gene. (XLSX)

S1 Fig The scheme of nested and anchored PCR. For nested PCR, brown arrows are external primers, dark blue arrows are internal primers. To design such four primers we should take into consideration the following conditions for external and internal primer: (1) non-target one primer hybridizations; (2) one primer pair non-target amplicons; (3) non-target amplicons for one pool primers (4) secondary structures between primers from the same and different amplicons. In case of primers with adapter sequences, we should take them into account while modeling secondary structures. For anchored PCR, we design one gene-specific primer that flanks highly variable region or region with unknown sequence (e.g. for gene fusions). We should consider (1) non-target one primer hybridizations and (2) secondary structures between one pool primers for different targets.
(TIF)

S2 Fig The graphical interface of the NGS-PrimerPlex program. Four different windows are shown (from left-top to right bottom): (1) main menu; (2) extraction of a gene(s)’ CDS coordinates; (3) primer design; (4) settings. Most settings have default values that can be used.
(TIF)

S3 Fig (**A**) Approach used by NGS-PrimerPlex, when for each position, the program calls primer3 to design three types of primers: so that the right primer was close to the studied position, the left primer was close to the studied position, and without any of these restrictions. Such type of primer design gives more flexibility on the next steps, when primer pairs are combined into sets of primers that amplify the whole studied region (e.g. exon). It is necessary because we can’t join two overlapping primer pairs into one multiplex reaction (**B**). x_i_, x_i+1_, x_i+2_ are genome positions placed one by one. L1.1 is a left primer of the first type for the first position; L1.2 is a left primer of the second type for the first position; R2.3 is a right primer of the third type for the second position etc. (**B**) Formation of non-target amplicon while joining of overlapping primer pairs into one multiplex reaction. At the same time, we need to design primers located one by one to read the whole sequence of the studied region, because after sequencing, for fragment L2-R2, only part denoted with asterisk make sense for calling variants. More information about the primers in the amplicon-based targeted NGS you can read in [22].
(TIF)
