# Supplementary figures and images for: NGS-PrimerPlex: High-throughput primer design for multiplex polymerase chain reactions
Source: PLoS Comput Biol. 2020 Dec 30;16(12):e1008468. doi: 10.1371/journal.pcbi.1008468 (PMC7802936; doi:10.1371/journal.pcbi.1008468)

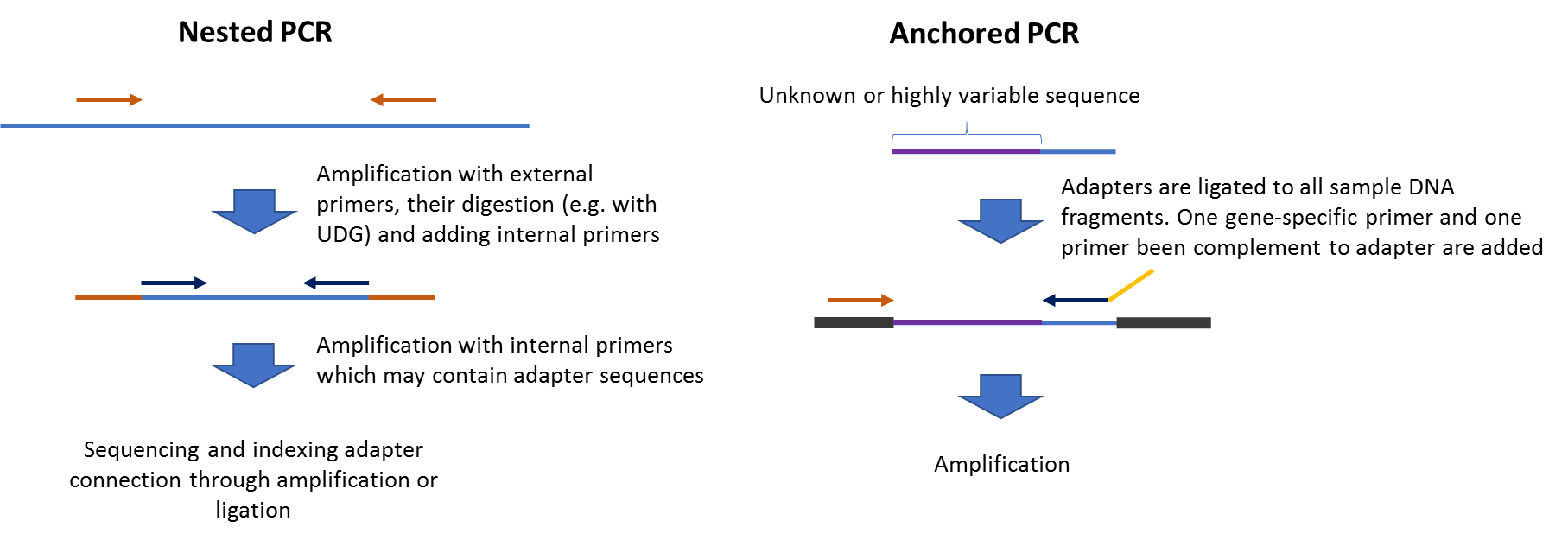

Supplement: S1 Fig — For nested PCR, brown arrows are external primers, dark blue arrows are internal primers. To design such four primers we should take into consideration the following conditions for external and internal primer: (1) non-target one primer hybridizations; (2) one primer pair non-target amplicons; (3) non-target amplicons for one pool primers (4) secondary structures between primers from the same and different amplicons. In case of primers with adapter sequences, we should take them into account while modeling secondary structures. For anchored PCR, we design one gene-specific primer that flanks highly variable region or region with unknown sequence (e.g. for gene fusions). We should consider (1) non-target one primer hybridizations and (2) secondary structures between one pool primers for different targets. (TIF) [file pcbi.1008468.s004.tif]

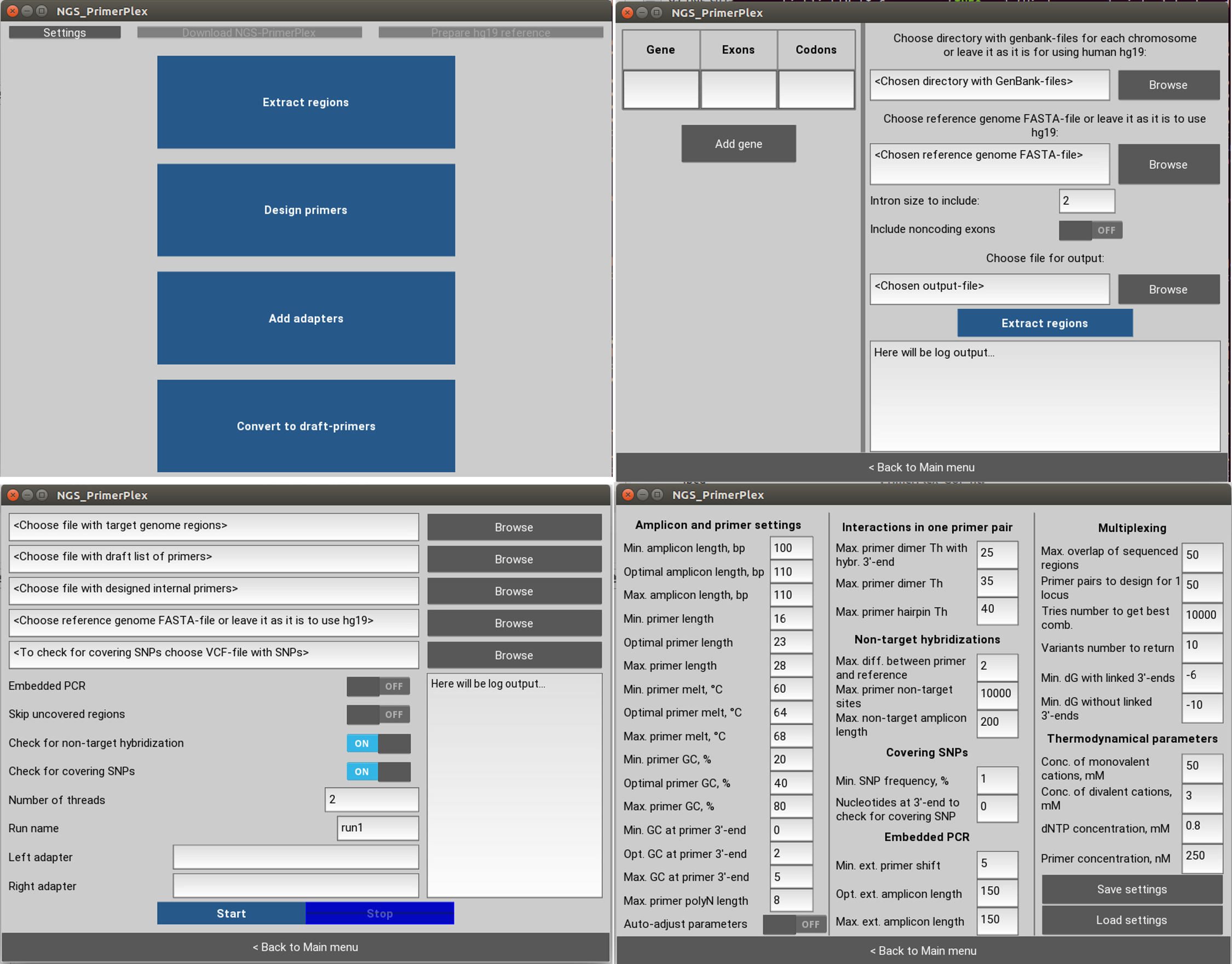

Supplement: S2 Fig — Four different windows are shown (from left-top to right bottom): (1) main menu; (2) extraction of a gene(s)’ CDS coordinates; (3) primer design; (4) settings. Most settings have default values that can be used. (TIF) [file pcbi.1008468.s005.tif]

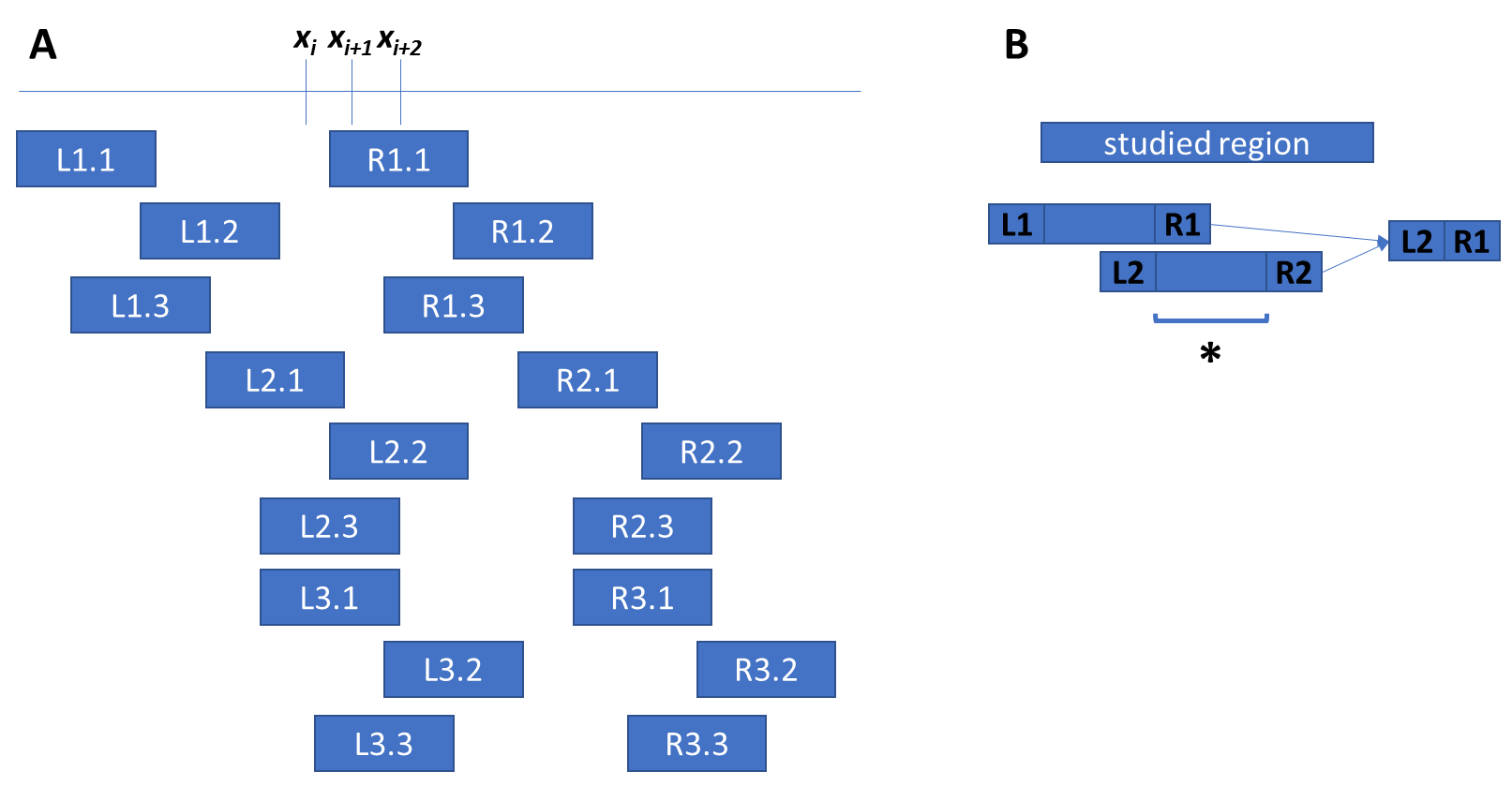

Supplement: S3 Fig — (A) Approach used by NGS-PrimerPlex, when for each position, the program calls primer3 to design three types of primers: so that the right primer was close to the studied position, the left primer was close to the studied position, and without any of these restrictions. Such type of primer design gives more flexibility on the next steps, when primer pairs are combined into sets of primers that amplify the whole studied region (e.g. exon). It is necessary because we can’t join two overlapping primer pairs into one multiplex reaction (B). xi, xi+1, xi+2 are genome positions placed one by one. L1.1 is a left primer of the first type for the first position; L1.2 is a left primer of the second type for the first position; R2.3 is a right primer of the third type for the second position etc. (B) Formation of non-target amplicon while joining of overlapping primer pairs into one multiplex reaction. At the same time, we need to design primers located one by one to read the whole sequence of the studied region, because after sequencing, for fragment L2-R2, only part denoted with asterisk make sense for calling variants. More information about the primers in the amplicon-based targeted NGS you can read in [22]. (TIF) [file pcbi.1008468.s006.tif]
